# Nitrous oxide (N2O) recreational use is increasing across Germany - a survey of the German neurological society among practicing neurologists

**DOI:** 10.1186/s42466-025-00425-9

**Published:** 2025-09-09

**Authors:** Johannes Heinrich Alexander Piel, Lucas Christoph Adam, Leona Möller, Peter Berlit

**Affiliations:** 1 Department of Neurology, University Hospital Schleswig-Holstein, Campus Kiel, Kiel, Germany; 2 Department of Neurology, Charité-Universitätsmedizin Berlin, Berlin, Germany; 3 Department of Neurology, Epilepsy Center Hessen, Philipps University Marburg, Marburg, Germany; 4 German Neurological Society, Berlin, Germany

**Keywords:** Nitrous oxide, N_2_O, Vitamin B12, Vitamin B12 deficiency, Subacute combined degeneration, Homocysteine, Public health, Thrombosis, Germany, Epidemiology

## Abstract

**Background:**

Recreational nitrous oxide (N_2_O) abuse has become increasingly prevalent, raising concerns about associated health risks. In Germany, the lack of reliable data on N_2_O consumption patterns limits the development of effective public health interventions. This study aims to address this knowledge gap by examining trends, determinants, and health consequences of N_2_O abuse in Germany.

**Methods:**

A two-phase online survey was conducted from July 17 to September 13, 2024 among members of the German Neurological Society (DGN). In the first phase (101 respondents), the frequency and trends of N_2_O-related cases were assessed. In the second phase (17 respondents) detailed information on diagnostic and therapeutic approaches was collected.

**Results:**

Occasional N_2_O-related cases were reported in 60% and 5% noted regular occurrences, particularly in the cities of Berlin and Frankfurt/Main. A nation-wide increase in case numbers was observed. Most neurologists treated between 1 and 10 cases annually, with metropolitan regions reporting higher numbers exceeding 30 per year. Myelopathy (94%) and neuropathy (88%) were widely recognized complications, whereas hypercoagulability (24%) and skin alterations (12%) were less frequently acknowledged. Vitamin B_12_ levels (94%) and differential blood counts (88%) were the most frequently assessed markers, while methylmalonic acid was most often regarded as the key parameter for detecting N_2_O-related vitamin B_12_ deficiency (78%). Treatment predominantly involved intramuscular vitamin B_12_ (88%), occasionally in combination with methionine (24%). Neurological deficits improved (median modified Rankin Scale score from 3 to 2), but 75% of cases relapsed after renewed N_2_O use.

**Conclusion:**

This study provides evidence of widespread N_2_O abuse in Germany, with hotspots in Berlin and Frankfurt/Main, and a concerning rise in rural areas. While myelopathy is well recognized among neurologists, other clinical manifestations are underreported. Improved communication, along with standardized diagnostics and treatment protocols, is urgently needed to address the heterogenous awareness of symptomatology, diagnostic sensitivity and specificity, and therapeutic strategies.

**Supplementary Information:**

The online version contains supplementary material available at 10.1186/s42466-025-00425-9.

## Background

Nitrous oxide (N_2_O), commonly known as ‘laughing gas’, is a volatile anaesthetic agent for medical use but is increasingly abused as a recreational drug, with the potential to cause severe neurological deficits. First synthesized in 1772 by Joseph Priestly, N_2_O gained popularity as a recreational inhalant in the late 18th century, prior to its medical application [1, 2]. Recent meta-analyses found the use of N_2_O increases the risk of pulmonary atelectasis in general anaesthesia, and its use has also recently been criticised for environmental considerations [3, 4]. Although its role in anaesthetics has declined, N_2_O continues to be widely employed as a propellant and foam agent (E942) in the production of whipped cream [2].

In recent years, however, N_2_O has re-emerged as a recreational drug, with consumption rising rapidly and poising significant public health concerns. Cases of N_2_O induced myeloneuropathy were first reported in 1978 among dentists who misused it [5]. Since then, a continued increase of both N_2_O abuse and neurological complications was observed. Notably, the Netherlands experienced a dramatic surge in N_2_O related cases between 2010 and 2020, from 6 to 144 cases per year [6]. In the United Kingdom, N_2_O has risen to the second most used drug and cases of N_2_O induced myeloneuropathy are increasingly reported in major cities such as London, Birmingham and Manchester [5]. According to a 2016 study, one in nine young Germans was estimated to have used N_2_O at least once in their lifetime. In the United Kingdom and the United States, one in three individuals reported past use [7]. Data from the North Rhine-Westphalia State Criminal Police Office indicated the number of consumers tripled within 2023 [8]. A 2025 multicentre study observed a similar increase from 2022 to 2024 [9], with comparable trends reported globally. In Australia, the number of users doubled in 2019 [10]. Particularly alarming is the shift from occasional use to high-frequency consumption. In 2016, only 4% of N_2_O users experienced neurological complications [7]. By 2024, this proportion had doubled [11].

N_2_O induces functional vitamin B_12_ deficiency by oxidizing its cobalt ion [12], thereby inactivating the vitamin B_12_ and impairing DNA synthesis and inducing defective formation of the myelin sheath, even in individuals normal baseline vitamin B_12_ levels. Routine testing may fail to detect this deficiency, making it imperative for healthcare providers to consider N_2_O exposure in patients presenting with corresponding symptoms. Early recognition and immediate initiation of high dose parenteral vitamin B_12_ therapy are crucial to prevent irreversible neurological damage [12].

Certain populations may be particularly vulnerable to N_2_O-induced neurological complications. Risk factors include dietary habits (e.g., veganism without supplementation), malnutrition, or gastrointestinal diseases (such as inflammatory bowel disease). Approximately 3.6% of adults have a severe vitamin B_12_ deficiency (< 200 pg/mL) and additional 12.5% have insufficient levels (< 300 pg/mL) [13]. Thus, about one in eight individuals may be especially vulnerable to N_2_O-associated neurological effects. In such individuals, as few as four inhaled balloons can result in paralysis, whereas some users consume 50 or more balloons in a single session [13].

The most common and severe complications include subacute combined degeneration of the spinal cord and peripheral neuropathy. Neurological symptoms are present in 89% of symptomatic users and most frequently include gait instability (57%), paresthesia (51%), numbness, and paralysis (each 31%). Autonomic nervous system involvement may lead to bladder dysfunction (7%) and impotence (2%). Psychiatric side effects, reported in 21% of users, include confusion (16%), hallucinations (8%), and mood disturbances (2%) [10].

Beyond neurological symptoms, vitamin B_12_ deficiency and the resulting elevation in homocysteine levels can impair coagulation, increasing the risk of thromboembolic events. Although less common, complications such as sinus and cerebral venous thrombosis, pulmonary embolism, deep vein thrombosis, myocardial infarction, and thrombosis of the liver and spleen have all been reported in N_2_O users [14]. Once spinal cord or peripheral nerve damage has occurred, paralysis or sensory deficits may persist lifelong despite optimal treatment [10].

This study aimed to assess the frequency of N_2_O-associated complications in Germany, as well as its associated symptoms, diagnostic approaches, and treatment strategies.

## Methods

The survey was conducted using a stepwise approach between 17 August and 13 September 2024, comprising two steps: Step one from 17 July to 14 August 2024, and step two from 14 August to 13 September 2024. All members of the German Neurological Society (DGN) were invited via email newsletter to participate in an online questionnaire assessing the incidence of N_2_O-related cases. In a census conducted on 30 August 2024, the DGN recorded 4,555 residents and 4,529 specialists. The newsletter was sent to 10,586 recipients including non-academics, of whom 128 (1.4% of neurologists contacted) opened the survey. To ensure anonymity, no demographic data was collected. However, the first two digits of the participants’ postal code were requested to guarantee a comprehensive geographic representation from across Germany. The original and translated versions of the questionnaire are available in the online supplementary (Supplementary Tables 1 and 2).

In the first step, participants were asked whether they had treated patients with neurological complications due to N_2_O use, whether these cases occurred regularly or only occasionally, whether the incidence had increased, decreased, or remained stable, and how many cases they had treated in the past year (Supplementary Table 1). In the second step, all respondents were invited to voluntarily complete a more detailed follow-up questionnaire (Supplementary Table 2). The questionnaire was iteratively developed and revised by local experts in N_2_O-related complications, based on previously published data on diagnostic and therapeutic practices.

The study was conducted in accordance with the Declaration of Helsinki and approved by local Ethics committee of the medical faculty of Kiel university (D 546/24).

Due to the low response rates in the second step, no inferential statistics were preformed; only descriptive statistics are reported.

## Results

**Fig. 1:**
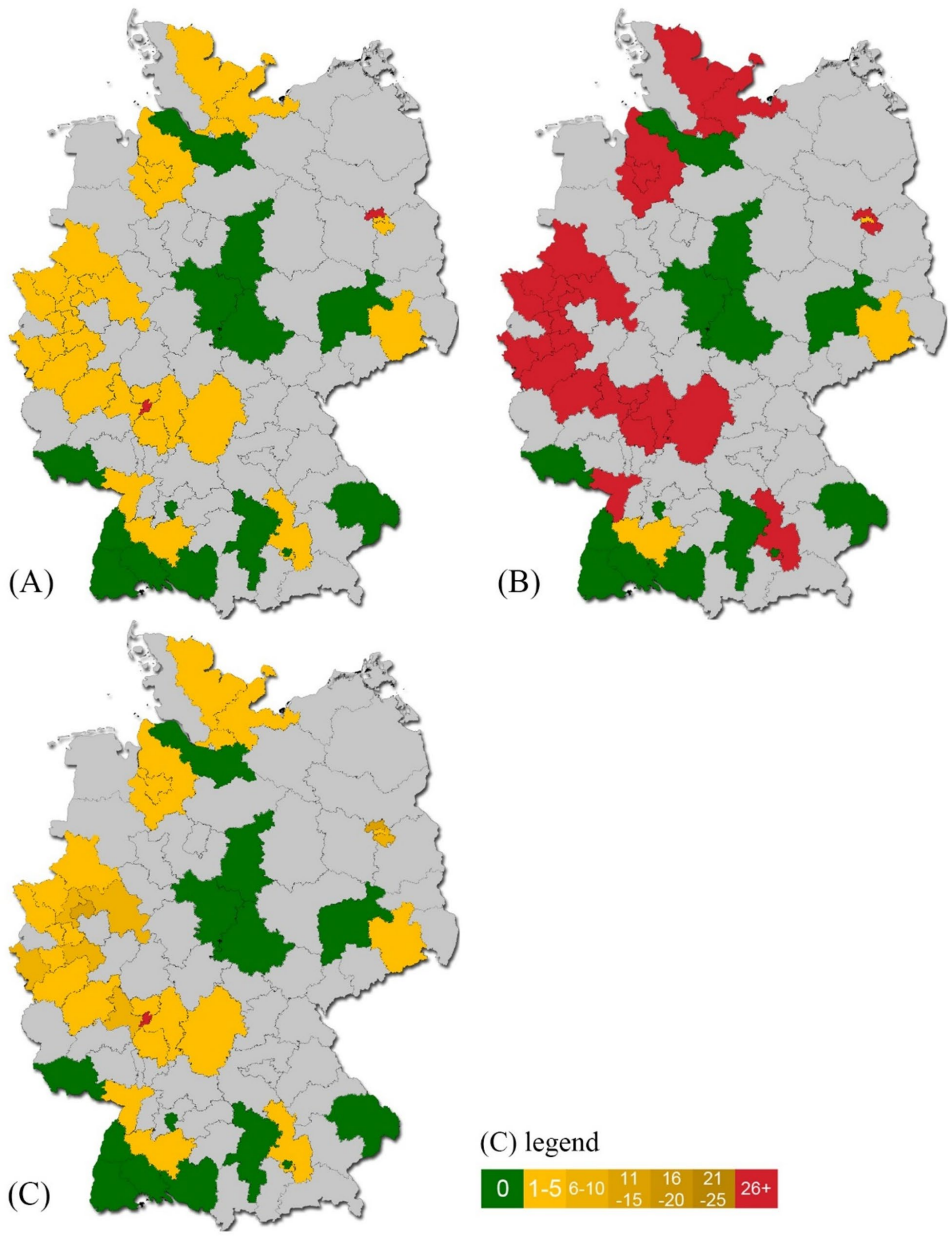
**A-C.** Responses to the questions **(A)** *‘How frequently have you treated patients with neurological complications due to recreational nitrous oxide use?’* Green: Never, Yellow: Occasionally, Red: Regularly, Grey: No data. **(B)** *‘How has the number of patients with neurological complications due to recreational nitrous oxide use changed in the past two years?’* Green: No patients, Yellow: No change, Red: Increase, Grey: No data. A decrease was not reported. **(C)** *‘How many patients with neurological complications due to recreational nitrous oxide use have been treated in your hospital in the past 12 months?’* Grey: No data

The first step of the survey was completed by 101 neurologists. Of these, 61 (60%) reported encountering neurological complications related to N₂O abuse occasionally, while 35 (35%) reported no such complications, and five neurologists (5%) from two identified hotspots, Berlin and Frankfurt/Main (Ffm), reported a regular occurrence of N_2_O-related neurological complications (Fig. 1A). A total of 60 (91% among those who reported cases) observed an increasing incidence, and 6 neurologists (9% among those who reported cases) noted a stable number of cases over the past two years (Fig. 1B). No neurologist reported a decrease of N_2_O-related complications. Most respondents treated between 1 and 10 cases per year, whereas hospitals in metropolitan areas reported higher case numbers, with Ffm exceeding 30 cases annually (Fig. 1C).

The second step (Tables 1 and 2) of the survey was completed by 17 neurologists (17% response rate). All participants in this step completed the whole questionnaire. Most respondents were specialists (15 out of 17), who estimated 5 (0–12) N_2_O capsules to suffice for neurological complications after 7 (0–16) days. The respondents worked at non-university hospitals (*n* = 8), at university hospitals (*n* = 7), or in private practice or medical clinics (*n* = 2). Myelopathy and neuropathy were known to 94% and 88% of respondents and blood count changes and encephalopathy to 76%. Hypercoagulability and skin alterations were only known to 24% and 12% respectively. A total of 82% and 71% observed myelopathy and neuropathy in their patients, 29% blood count changes and 12% encephalopathies. No hypercoagulability or skin alterations were reported.

Myeloneuropathy was treated by 88% with intramuscular vitamin B_12_, with additional methionine in 24%. Oral vitamin B_12_ treatment was initiated in 12%. Modified Rankin Scale (mRS) presentation improved from 3 ± 1 (median ± IQR) on admission to 2 ± 1 at discharge. The recurrence rate after renewed N_2_O abuse was 75%.

Vitamin B_12_ levels (94%) and differential blood counts (88%) were the most frequently assessed parameters in patients with suspected N_2_O-related complications, followed by methylmalonic acid (MMA) (71%), folic acid (71%), holotranscobalamin (65%), and homocysteine (53%). Participants considered methylmalonic acid the most reliable diagnostic marker (78%), followed by spinal cord MRI (69%) and vitamin B_12_ levels (32%). Focussed medical history taking was considered the most important diagnostic tool (53%), followed by blood parameters (26%) and spinal cord MRI (12%). Expectations regarding electrophysiology findings were heterogenous: 76% of respondents anticipated pathological somatosensory evoked potentials (SSEP), 59% expected signs of peripheral axonal damage, 53% peripheral demyelination, 24% pathological motor evoked potentials (MEP), 24% pathological spontaneous activity in electromyography, and 18% chronic neurogenic remodelling.

**Table 1:** Observations in outpatient practice/medical care centers, non-university and university hospitals. mRS = modified Rankin scale. N_2_O = nitrous oxide. Median ± IQR

	Practice/ medical care center	Non-university hospital	University hospital	Total
**Participating neurologists** Residents Specialists	2 0 2	8 0 8	7 2 5	**17** **2** **15**
**Cases / 12 months**	1.5 ± 0.5	1.5 ± 2	2 ± 2	**2** ± **2**
**Estimated number of N**_**2**_**O capsules (**≙** 8 g) inducing neurological sequelae**	10.5 ± 9.5	6.5 ± 10.75	5 ± 1.5	**5** ± **7**
**Estimation of delay (days) between N**_**2**_ **O consumption and neurological symptoms**	5.5 ± 0.5	7 ± 7.5	14 ± 10	**7** ± **9**
**Symptoms known**				
Myelopathy Neuropathy Hypercoagulability Red blood cell count Encephalopathy Skin alterations	2 (100%) 1 (50%) 1 (50%) 1 (50%) 1 (50%) 1 (50%)	8 (100%) 7 (88%) 1 (13%) 6 (75%) 7 (88%) 0 (0%)	6 (86%) 7 (100%) 2 (29%) 6 (86%) 5 (71%) 1 (14%)	**16 (94%)** **15 (88%)** **4 (24%)** **13 (76%)** **13 (76%)** **2 (12%)**
**Symptoms observed**				
Myelopathy Neuropathy Hypercoagulability Red blood cell count Encephalopathy Skin alterations	2 (100%) 1 (50%) 0 (0%) 1 (50%) 0 (0%) 0 (0%)	7 (88%) 6 (75%) 0 (0%) 2 (25%) 1 (13%) 0 (0%)	5 (71%) 5 (71%) 0 (0%) 2 (29%) 1 (14%) 0 (0%)	**14 (82%)** **12 (71%)** **0 (0%)** **5 (29%)** **2 (12%)** **0 (0%)**
**Treatment reported**				
Vitamin B_12_ p.o. Vitamin B_12_ i.m. Methionin p.o.	1 (50%) 1 (50%) 0 (0%)	1 (13%) 8 (100%) 3 (38%)	0 (0%) 6 (86%) 1 (14%)	**2 (12%)** **15 (88%)** **4 (24%)**
**mRS at presentation** **mRS after treatment**	3.5 ± 0,5 1.5 ± 0,5	3 ± 1 2 ± 0,25	3 ± 1 2 ± 1	**3** ± **1** **2** ± **1**
**Estimation of myelo- and neuropathy recovery rate**	75% ± 5.5%	44% ± 24%	70% ± 25%	**61%** ± **27%**
**Estimation of recurrences after repeat of N**_**2**_ **O abuse**	72% ± 8%	75% ± 15%	75% ± 33%	**75%** ± **28%**

**Table 2:** Findings and estimated predictive value of diagnostic tools in recreational nitrous oxide consumption. PSA = Pathological spontaneous activity. SSEP = Somatosensory evoked potentials. MEP = Motor evoked potentials. Median ± IQR

	Practice/ medical care center	Non-university hospital	University hospital	Total
**Lab parameters tested**				
Blood count Differential blood count Vitamin B_12_ Holotranscobalamine Methylmalonic acid Folic acid Homocysteine	0 (0%) 2 (100%) 2 (100%) 2 (100%) 1 (50%) 1 (50%) 2 (100%)	5 (63%) 7 (88%) 8 (100%) 4 (50%) 7 (88%) 7 (88%) 3 (38%)	3 (43%) 6 (86%) 6 (86%) 5 (71%) 5 (71%) 4 (57%) 4 (57%)	**8 (47%)** **15 (88%)** **16 (94%)** **11 (65%)** **13 (76%)** **12 (71%)** **9 (53%)**
**Perceived reliability**				
Vitamin B_12_ Methylmalonic acid Spinal cord MRI	25% ± 25% 70% ± 20% 82% ± 2,5%	29% ± 32% 78% ± 21% 66% ± 24%	34% ± 23% 82% ± 8% 69% ± 18%	**32%** ± **31%** **78%** ± **15%** **69%** ± **24%**
**Parameter expected for highest specificity**				
Medical history Clinical examination Blood parameters Electrophysiology Spinal cord MRI	1 (50%) 1 (50%) 0 (0%) 0 (0%) 0 (0%)	4 (50%) 0 (0%) 2 (25%) 0 (0%) 2 (25%)	4 (57%) 0 (0%) 2 (29%) 1 (14%) 0 (0%)	**9 (53%)** **1 (6%)** **4 (26%)** **1 (6%)** **2 (12%)**
**Electrophysiological findings**				
No changes Light demyelinisation Severe demyelinisation Light axonal damage Severe axonal damage PSA Chronic remodelling Pathological SSEP Pathological MEP	0 (0%) 0 (0%) 0 (0%) 0 (0%) 1 (50%) 1 (50%) 0 (0%) 2 (100%) 1 (50%)	0 (0%) 3 (38%) 2 (25%) 2 (25%) 3 (38%) 2 (25%) 1 (13%) 7 (88%) 2 (25%)	0 (0%) 1 (14%) 4 (57%) 2 (29%) 2 (29%) 1 (14%) 2 (29%) 4 (57%) 1 (14%)	**0 (0%)** **4 (24%)** **6 (35%)** **4 (24%)** **6 (35%)** **4 (24%)** **3 (18%)** **13 (76%)** **4 (24%)**

## Discussion

Our study contributes to the growing body of evidence on the increasing prevalence of patients with N_2_O-induced complications seeking medical attention in Germany. We identified two hotspots in Berlin and Ffm but also found indications of widespread abuse across the country. This is consistent with data showing a general increase in substance abuse in these cities [15]. Furthermore, we provided evidence that myeloneuropathy is recognized by German neurologists, while rarer non-neurological presentations, such as hematological complications, may be overlooked.

The data on N_2_O abuse in urban hotspots align closely with reports from other European capitals [5, 11] and densely populous cities such as Ffm [16]. Although N_2_O misuse has predominantly been reported in urban settings [7, 17], the findings also indicate a notable prevalence in rural areas, where retail availability (e.g. corner shops) is limited or non-existent. This pattern suggests that unrestricted online access to large containers plays a critical role [9, 17, 18]. This highlights the need for consumer warnings and suggests that urgent legislative action may be required to reduce N_2_O abuse in Germany. The estimate of neurological sequelae occurring after the consumption of five N₂O capsules (≈ 8 g) with a latency of seven days may represent a slight overestimation of both the required dose and the time to symptom onset when compared with data from a recent case series [19]. In that study, neurological symptoms typically emerged after a median of 10 weeks of daily N_2_O use, whereas in cases of extreme consumption (e.g., > 1000 capsules per day), symptoms developed within less than one month. However, the literature on the latency between last N_2_O use and onset of neurological symptoms remains scarce.

According to survey responses, German neurologists have substantial expertise in N_2_O and related vitamin B_12_ metabolism and routinely administer high dose vitamin B_12_ for patients with N_2_O abuse. German neurologists prefer treatment by intramuscular B_12_ injections for those patients, however, evidence suggests that high dose intramuscular vitamin B_12_ supplementation is equally effective as oral administration in increasing serum B_12_ levels [20]. Methionine and folic acid have been proposed as potential adjunct therapies; however, there is no clinical evidence supporting their routine use [21]. To date, no consensus has been established regarding the treatment regimen for neurological sequelae following N_2_O abuse. Nonetheless, high dose vitamin B_12_ administration and cessation of N_2_O exposure remain the clinical standard [22].

Most survey respondents primarily rely on serum B_12_ levels for diagnosing N_2_O-induced B_12_ deficiency, with MMA and homocysteine being used less frequently despite its higher sensitivity in detecting functional B₁₂ deficiency after N_2_O abuse. A recent German multicenter study on neurological complications after N_2_O abuse reported a low association of symptom presentation and serum B_12_ levels (35%), whereas MMA (95%) and homocysteine (89%) were highly sensitive markers for N_2_O-induced B₁₂ deficiency [9, 16]. These findings align with a 2019 systematic review, which reported similar sensitivity values (serum B_12_: 71%, MMA: 94%, homocysteine: 90%) [23]. N_2_O does not directly lower serum B_12_ levels but inactivates cobalamin, leading to functional B_12_ deficiency. Holotranscobalamin, the biologically active form of vitamin B_12_, may not reliably distinguish between functional and dysfunctional cobalamin [23]. Therefore, both absolute serum B_12_ levels and holotranscobalamin have limited clinical utility as biomarkers in the context of N_2_O abuse.

While MMA and homocysteine are strong indicators of detecting N_2_O-induced B_12_ deficiency, their measurement was less commonly performed by clinicians in this survey. Potential reasons for this include the relatively high costs associated with MMA testing, the preanalytical thermal instability of homocysteine, and insufficient awareness of the most reliable biomarkers for B_12_ deficiency detection. Recent studies have linked MMA levels to clinical severity [24] and the cumulative amount of N_2_O intake [5], suggesting that MMA may have the highest biochemical and clinical sensitivity for assessing B_12_ deficiency caused by N_2_O abuse.

Most survey respondents treated their patients with intramuscular B_12_. This is in accordance with the recommendation to start B_12_ treatment with 1000 µg intramuscularly daily for 1–2 weeks, followed by 1000 µg intramuscular weekly or 2000 µg oral daily until resolution of symptoms [21, 25]. When treating patients with neurological sequelae from N_2_O abuse German neurologists observed clinical improvement, progressing from moderate to slight disability, though residual symptoms frequently remained at discharge. This aligns with a recent case series that followed five patients and found that neurological sequelae could persist in individual cases even after ten years [19]. Given the presumably increasing prevalence of N_2_O abuse in Germany and the current lack of restrictions, further awareness among clinicians is essential.

Clinicians consider medical history taking to have the highest diagnostic value – followed by clinical examination, laboratory assessment, and imaging or electrophysiological studies. Identifying a history of N_2_O abuse is crucial for the diagnosis [5], especially as other causes of B_12_ deficiency may also present with varying degrees of myelopathy and peripheral neuropathy [26]. Furthermore, non-B_12_-associated differential diagnoses, such as Guillain-Barré syndrome, may also present with symptoms such as gait disturbances and ascending paresthesias. A thorough medical history can, prior to any other diagnostic steps, facilitate the initiation of therapy, such as B_12_ substitution, or eliminate the need for unnecessary invasive procedures, such as a lumbar puncture [27].

Expectations regarding electrophysiological findings in N_2_O-induced B_12_ deficiency are consistent with real-world clinical data [9, 19]. In line with previous research, survey respondents assumed that spinal MRI has low specificity for diagnosing this condition [28]. However, spinal MRI can still be valuable in assessing the extent of myelopathy, determining the severity of sequelae, and serving as a tool for follow-up diagnostics [19].

### Limitations

The main limitation of this study was a low response rates with 17% respondents in step two, facilitating potentially a bias towards neurologists who are interested in this topic. The study is not representative due to low response and possible selection bias. Furthermore, responders were not equally distributed throughout Germany with significant high responding rates in Berlin and Ffm. Further research is needed to determine whether these clusters were reported as a cause of higher drug abuse or more awareness of N_2_O complications. One further limitation lies in the retrospective self-reporting nature of the data, which can be prone to various biases. Participants may not accurately recall past events or experiences, and their responses may be influenced by recent occurrences.

## Conclusion

Overall, this paper serves as a comprehensive resource on the ongoing debate regarding neurological sequelae following N_2_O abuse in Germany. It provides a foundation for increasing clinical awareness, fostering further investigation into policy solutions. German neurologists report a growing number of patients presenting with neurological complications related to N_2_O abuse in clinical practice. This survey highlights the potentially severe and lasting neurological consequences of N_2_O abuse within the context of its unregulated distribution in Germany, underscoring the need for preventive regulations and increased awareness among N_2_O users. The variability in diagnostic approaches and treatment regimens among experts emphasizes the necessity of future guidelines to streamline clinical management.

## Supplementary Information

Below is the link to the electronic supplementary material.

Supplementary Material 1

## Data Availability

The datasets used and/or analysed during the current study are available from the corresponding author on reasonable request but restrictions may apply on data that may compromise anonymity of participants. The data are not publicly available due to privacy or ethical restrictions.

## Abbreviations

DGN: German Neurological Society
Ffm: Frankfurt am Main
MEP: Motor evoked potentials
MMA: Methylmalonic acid
MRI: Magnet resonance imaging
mRS: Modified Rankin Scale
N_2_O: Nitrous oxide
PSA: Pathological spontaneous activity
SSEP: Somatosensory evoked potentials
