# Supplementary material for: Nitrous oxide (N2O) recreational use is increasing across Germany - a survey of the German neurological society among practicing neurologists
Source: Neurol Res Pract. 2025 Sep 9;7(1):64. doi: 10.1186/s42466-025-00425-9 (PMC12421744; doi:10.1186/s42466-025-00425-9)
Supplement: Supplementary file 1 — Supplementary Material 1 [file 42466_2025_425_MOESM1_ESM.docx]

**Supplementary Material**

**Supplementary Table 1.** Survey part 1 in English (translation) and German (original)

| **Question** | **Options** |
| --- | --- |
| *How frequently have you treated patients with neurological complications due to recreational nitrous oxide use?* | - Never - Occasionally - Regularly |
| *How has the number of patients with neurological complications due to recreational nitrous oxide use changed in the past two years?* | - Increase - No change - Decrease |
| *How many patients with neurological complications due to recreational nitrous oxide use have been treated in your hospital in the past 12 months?* | Free-text number |
| *In which postcode do you practise clinically?* | _ _ X X X (first two digits of the postal code) |
| **Frage** | **Antwortoptionen** |
| *Wie oft werden in Ihrer Klink Patient:innen aufgrund der gesundheitlichen Folgen eines Lachgaskonsums behandelt?* | - Nie - Gelegentlich - Regelmäßig |
| *Hat sich in den letzten zwei Jahren in Ihrer Klinik die Zahl der Patient:innen, die aufgrund der gesundheitlichen Folgen eines Lachgaskonsums behandelt wurden, verändert?* | - Zugenommen - Keine Veränderung - Abgenommen |
| *Wie viele Patient:innen wurden in den vergangenen 12 Monaten in Ihrer Klinik aufgrund der gesundheitlichen Folgen eines Lachgaskonsums behandelt?* | Freitextzahl |
| *In welchem PLZ-Gebiet sind Sie tätig?* | _ _ X X X (ersten zwei Ziffern der Postleitzahl) |

**Supplementary Table 2.** Survey part 2 in English (translation) and German (original)

| **Question** | **Options** |
| --- | --- |
| *How many cases of nitrous oxide use have you treated in the last 12 months?* | Free-text number |
| *What are the consequences of nitrous oxide consumption that you are aware of?* | - Myelopathy - Neuropathy - Hypercoagulability - Blood-count changes - Encephalopathy - Skin alterations - Other (free-text) |
| *What consequences of nitrous oxide consumption have you observed in your patients?* | - Myelopathy - Neuropathy - Hypercoagulability - Blood-count changes - Encephalopathy - Skin alterations - Other (free-text) |
| *Which laboratory parameters do you routinely measure when nitrous oxide myeloneuropathy is suspected?* | - Blood-count - Differential blood-count - Vitamin B12 - Holotranscobalamine - Methylmalonic acid - Folic acid - Homocysteine - Other (free-text) |
| *In your opinion, which diagnostic method has the highest specificity in nitrous oxide myeloneuropathy diagnosis?* | - Medical history - Neurological examination - Blood parameters - Electrophysiology - Spinal MRI - Other (free-text) |
| *What electrophysiological findings do you expect in nitrous oxide myeloneuropathy?* | - None of the below - Light demyelinating neuropathy - Severe demyelinating neuropathy - Light axonal neuropathy - Severe axonal neuropathy - Myopathy - Chronic-neurogenic changes - Pathological spontaneous activity - Pathological somatosensory evoked potentials - Pathological motor evoked potentials |
| *How high do you estimate the sensitivity of vitamin B12 levels for nitrous oxide myeloneuropathy?* | 0-100% |
| *How high do you estimate the sensitivity of methylmalonic acid levels for nitrous oxide myeloneuropathy?* | 0-100% |
| *How high do you estimate the sensitivity of spinal MR tomography for nitrous oxide myeloneuropathy to be?* | 0-100% |
| *How high do you estimate the recovery rate of paresis in nitrous oxide myeloneuropathy under high-dose vitamin B12 supplementation?* | 0-100% |
| *How high do you estimate the recurrence rate to be in the event of renewed nitrous oxide consumption after successful treatment?* | 0-100% |
| *After how many cartridges can neurological complications be expected?* | Free-text number |
| *What do you estimate: On average, how long after the last inhalation of nitrous oxide do the first symptoms appear?* | Free-text number |
| *How would you rate the average degree of disability of the patients presenting before and after therapy using the modified Rankin Scale (0 = no symptoms, 6 = death)?* | - mRS at admission - mRS at discharge |
| *How do you treat your patients?* | - Oral vitamin B12 - Intramuscular vitamin B12 - Methionine - Other (free-text) |
| *In which postcode do you practise clinically?* | _ _ X X X (first two digits of the postal code) |
| *What level of further education do you have?* | - Resident - Neurology Specialist |
| *Where do you practice?* | - Practice / Medical care center - Non-university hospital - University hospital - Other (free-text) |
| *How many neurological physicians work at your location?* | Free-text number |
| **Frage** | **Antwortoptionen** |
| *Wie viele Fälle von Lachgas-Konsum haben Sie in den letzten 12 Monaten behandelt?* | Freitextzahl |
| *Welche Folgeerscheinungen von Lachgas-Konsum kennen Sie?* | - Myelopathie - Neuropathie - Gerinnungsstörung - Blutbildveränderungen - Enzephalopathie - Hautveränderungen - Anderes (Freitext) |
| *Welche Folgeerscheinungen nach Lachgas-Konsum haben Sie bei Ihren Patient*innen gesehen?* | - Myelopathie - Neuropathie - Gerinnungsstörung - Blutbildveränderungen - Enzephalopathie - Hautveränderungen - Anderes (Freitext) |
| *Welche Laborparameter erheben Sie regelhaft bei Verdacht auf eine Lachgas-Myeloneuropathie?* | - Blutbild - Differenzialblutbild - Vitamin B12 - Holotranscobalamin - Methylmalonsäure - Folsäure - Homocystein - Anderes (Freitext) |
| *Welche Diagnostik hat Ihrer Ansicht nach die höchste Spezifität bei der Diagnosefindung?* | - Anamnese - Klinisch-neurologische Untersuchung - Blutparameter - Elektrophysiologie - Spinales MRT - Anderes (Freitext) |
| *Welche elektrophysiologischen Befunde erwarten Sie?* | - Keine der genannten - Leichte demyelinisierende Neuropathie - Schwere demyelinisierende Neuropathie - Leichte axonale Neuropathie - Schwere axonale Neuropathie - Myopathische Veränderungen im EMG - Chronisch-neurogene Veränderungen im EMG - Pathologische Spontanaktivität im EMG - Pathologische SSEP - Pathologische MEP |
| *Wie hoch schätzen Sie die Sensitivität des Vitamin-B12-Spiegels für eine Lachgas-Myeloneuropathie ein?* | 0-100% |
| *Wie hoch schätzen Sie die Sensitivität des Methylmalonsäure-Spiegels für eine Lachgas-Myeloneuropathie ein?* | 0-100% |
| *Wie hoch schätzen Sie die Sensitivität der spinalen MR-Tomographie für eine Lachgas-Myeloneuropathie ein?* | 0-100% |
| *Wie hoch schätzen Sie die Rückbildungsquote von Paresen bei Lachgas-Myeloneuropathie unter hochdosierter Vitamin-B12-Substitution ein?* | 0-100% |
| *Wie hoch schätzen Sie die Rezidivrate bei einem erneuten Lachgas-Konsum nach erfolgreicher Behandlung ein?* | 0-100% |
| *Ab welcher Anzahl von Kartuschen ist mit neurologischen Komplikationen zu rechnen?* | Freitextzahl |
| *Was schätzen Sie: Mit welcher Latenz treten nach letztmaliger Lachgas-Inhalation im Durchschnitt die ersten Beschwerden auf?* | Freitextzahl |
| *Wie schätzen Sie den durchschnittlichen Grad der Behinderung der sich vorstellenden Patient*innen vor und nach Therapie anhand der modified Rankin Scale (0 = keine Symptome, 6 = Tod) ein?* | - mRS bei Erstvorstellung - mRS nach Therapie |
| *Wie behandeln Sie Ihre Patient*innen?* | - Vitamin B12 oral - Vitamin B12 i.m. - Methionin - Sonstiges (Freitext) |
| *In welcher Postleitzahl sind Sie klinisch tätig?* | _ _ X X X (erste zwei Ziffern der Postleitzahl) |
| *Welchen Weiterbildungsstand haben Sie?* | - Arzt/Ärztin in Weiterbildung - Fachärztin/Facharzt für Neurologie |
| *Wo sind Sie klinisch tätig?* | - Praxis, MVZ - Nicht universitäres Krankenhaus - Universitäres Krankenhaus - Anderes (Freitext) |
| *Wie viele neurologische ärztliche Mitarbeiter*innen sind an Ihrem Standort tätig?* | Freitextzahl |
